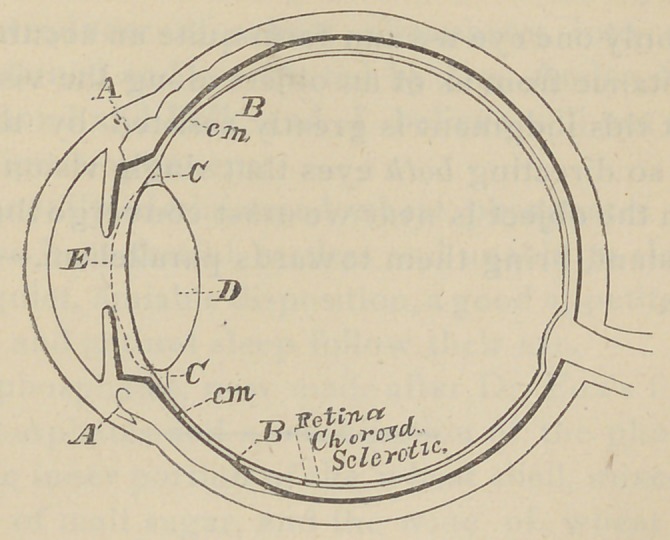# How We Judge Distances

**Published:** 1879-06

**Authors:** C. H. Stowell

**Affiliations:** Physiological Laboratory, University of Mich.


### Miscellaneous.

### HOW WE JUDGE DISTANCE.

BY C. H. STOWELL, M. D., PHYSIOLOGICAL LABORATORY, UNIVERSITY OF MICH.

If anyone desires to ascertain the weight of a body, he does not simply let the body press on the hand as it lies flat on the table, but holds it in the hand, lifts it up and down, and by the amount of muscular exertion required to support it and move it, forms an accurate judgment of its weight.

The consciousness then is more than one of pressure alone. There is a certain amount of muscular contraction. He is aware what muscles are thrown into action and to what extent. He is conscious of a " muscular sense." This " sense " is felt not only when the muscles are made to contract by the force of the will, but also when they contract under stimulation of the galvanic current. A strong argument in favor of the peripheral origin of this " sense."

We believe it is owing largely to this sense that we are able to judge of the distance between us and objects more or less remote.

That we may better understand this, let us turn to the mechanism of accommodation; for in order that objects both near and distant may be seen with equal distinctness, it is necessary that either the distance between the lens and the retina be changed, or that the refractive power of the lens be changed.

If we direct our attention from an object a long distance from us to one near at hand we are certainly conscious of some change taking place in the eye. If, again, we look at an object afar off, the change is not one of effort as before, but relief; due to the fact that in its quiescent state the eye is adjusted for distance. What is this effort, this change occurring in the eye when changing suddenly from a far to a near object? During accommodation for near objects two changes occur, and only two, of any importance.

First, contraction of the pupil; and second, change in the shape of the lens.

The first is simply to cut off the more divergent rays of light and is of but little influence in the formation of the image.

The second change is that the anterior surface of the lens becomes more convex. This need not be discussed, as no doubt is entertained concerning it.

How is this change brought about? We answer: By the action of the ciliary muscle. When the eye is adjusted for distant objects the suspensory ligament holds the lens so tense that its anterior surface is flattened as seen in the accompanying cut.

When adjusted for near objects the ciliary muscle now relaxed and at rest, contracts, which pulls forward the choroid coat and thus slackens the tense suspensory ligament. Now, owing to the elasticity of the lens, it bulges forward and thus its anterior surface is made more convex.

In 1868, Hensen and Voelcher, actually saw the choroid drawn forward during accommodation. We know most certainly that the lens is elastic and when taken from the body its anterior surface is more convex than when in its normal position in the body. We will take for the origin of this muscle, mainly from the line of union of the cornea with the sclerotic ; its insertion is lost in the choroid a trifle back of the anterior limit of the retina. Let *A* represent the origin and *B* the insertion of this muscle. The muscle is now relaxed, the suspensory ligament is tense and the anterior surface of the lens flattened.

Let this muscle contract.

Let *B* be brought nearer to *A*.

The suspensory ligament *C* will be relaxed and the lens *D* will bulge forward to the dotted line *E*.

If the object is near us the muscle will contract with a certain amount of force. If nearer it must contract still more and the "muscular sense" of the effort will enable us to tell whether the object is near at hand or far away. *A certain amount of muscular effort being associated with a certain distance.*

With only one eye we can form quite an accurate judgment of the distance from us of an object along the visual axis ; yet no doubt this judgment is greatly assisted by the muscular effort of so directing *both* eyes that single vision shall result ; for when the object is near we must converge the visual axes, and if distant, bring them towards parallelism.—*The Medical Advance.*

## Figures and Tables

**Figure f1:**